# Thermal Atoms Facilitate Intensity Clipping Between Vectorial Dual‐Beam Generated by a Single Metasurface Chip

**DOI:** 10.1002/advs.202416617

**Published:** 2025-03-07

**Authors:** Chen Qing, Jialong Cui, Lishuang Feng, Dengke Zhang

**Affiliations:** ^1^ School of Instrumentation and Optoelectronic Engineering Beihang University Beijing 100191 China

**Keywords:** beam shaping, light–atom interaction, metasurfaces, thermal atoms, vector beams

## Abstract

Manipulating vector beams is pivotal in fields such as particle manipulation, image processing, and quantum communication. Flexibly adjusting the intensity distribution of these beams is crucial for effectively realizing these applications. This study introduces a vectorial dual‐beam system utilizing thermal atoms as the medium for modulating the intensity profile of vector beams. A single metasurface is employed to generate both the control and signal vector beams, each with unique vectorial characteristics. The shaping of the signal beam profile is facilitated by the interaction with thermal atoms, which can be controlled by adjusting the control vector beam. This spatially selective absorption is a result of the thermal atoms' response to the varying polarizations within the vector beams. In this experiment, two distinct metasurface chips are fabricated to generate vector beams with doughnut‐shaped and Gaussian‐shaped intensity profiles. By adjusting the incident power and polarization state of the control light, the doughnut‐shaped signal beams can be converted into a rotational dual‐lobed pattern or the dimensions of the Gaussian‐distributed signal beams can be modified. This study introduces a novel vector beam shaping technique by integrating metasurfaces with thermal atoms, offering significant promise for future applications requiring miniaturization, dynamic operation, and versatile control capabilities.

## Graphical abstract

A vectorial dual‐beam system, comprising control and signal beams generated by a single metasurface chip and combined with thermal atoms, offers an innovative method for facilitating intensity clipping. By adjusting the incident power and polarization state of the control light, the intensity distribution of the signal beams can be significantly modulated.

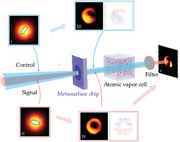

## 1 Introduction

Vector beams feature an inhomogeneous polarization distribution across their cross‐section, offering enhanced flexibility for the spatial manipulation and control of light fields.^[^ ^1^, ^2^ ^]^ This characteristic enables their widespread use in various applications, including optical manipulations,^[^ ^3^, ^4^, ^5^, ^6^ ^]^ optical communications,^[^ ^7^, ^8^, ^9^, ^10^, ^11^ ^]^ and sensing technologies.^[^ ^12^, ^13^ ^]^ Vector beams are conventionally generated through the use of wave plates, spatial light modulators, or digital micromirror devices. Although these approaches facilitate the manipulation of light fields, they also impose certain constraints and augment system complexity. In recent years, metasurface technology has made considerable progress, greatly improving the ability to manipulate light, especially in the field of shaping vector beams. Advanced micro‐nano fabrication techniques have enabled the creation of nanoscale meta‐atoms in metasurfaces, thereby allowing for precise manipulation of the polarization and intensity distributions of vector beams.^[^ ^14^, ^15^, ^16^, ^17^, ^18^, ^19^ ^]^ The application of metasurfaces in vector beam manipulation presents significant potential for advancements in optical tweezers^[^ ^20^ ^]^ and image processing.^[^ ^21^, ^22^, ^23^, ^24^, ^25^ ^]^ To facilitate the dynamic modulation of vector beams and enhance their manipulation, thereby enabling the investigation of diverse physical phenomena and broadening their potential applications, it is imperative to implement innovative control mechanisms. Recent advancements have demonstrated that exploiting light‐atom interactions presents a promising approach for the control of vector beams.^[^ ^26^ ^]^

In the context of light‐atom interactions, the state of atoms is altered by light, while simultaneously, atoms modify their properties in response to the light. This process involves transitions between different atomic energy levels through the absorption or emission of photons, with the probability of these transitions being governed by selection rules. Generally, the atomic response exhibits heightened sensitivity to the polarization state of the incident light. Variations in polarization states result in distinct atomic responses, thereby imparting a pronounced polarization dependence to the absorption process. Consequently, the utilization of atomic responses to vector beams has garnered extensive investigation across various domains, including magnetic field sensing,^[^ ^27^, ^28^, ^29^ ^]^ optical spatial mode extraction^[^ ^30^, ^31^, ^32^ ^]^ and optical storage.^[^ ^33^ ^]^ Nevertheless, in the present scenario of vector beam‐atom interactions, vector beams are predominantly produced with traditional optical elements, which restricts the diversity of vector beams configurations that can be achieved.

Additionally, directly manipulating input signal parameters to control vector beams may disrupt the stability of the optical path. Therefore, incorporating additional physical fields to modulate the signal light proves to be significantly more effective. This modulation can be achieved by interacting with atoms using magnetic fields,^[^ ^34^, ^35^, ^36^ ^]^ electric fields,^[^ ^37^ ^]^ or light fields.^[^ ^38^ ^]^ Among various methodologies, the use of a light field to shape another light field is particularly noteworthy due to its exceptional efficiency, simplicity, and minimal noise. This approach offers significant advantages over the generation of magnetic and electrical fields, as well as the complexities associated with assembling experimental setups.

In this paper, we propose a metasurface capable of generating vector beams that exhibit a variety of polarization states along their beam profiles. A vectorial dual‐beam configuration has been developed, consisting of control and signal beams that propagate concurrently in the same direction through the metasurface chip. By incorporating a thermal atomic vapor cell into the setup, a system is established where the vectorial dual‐beam engages with an atomic ensemble, allowing for precise manipulation of the signal beam's intensity distribution. In this arrangement, the incident signal light remains unchanged, and the output signal beam's intensity profile is modulated by varying the power or polarization state of the control light. This facilitates a system where the signal beam is shaped or tailored by the control beam. In the experiment, we designed and fabricated two metasurface chips capable of generating vector beams with distinct polarizations and intensity distributions. These chips were incorporated into the system to demonstrate its capability in regulating various vector beams configurations. Our approach capitalizes on the distinctive sensitivity of thermal atoms to vector beams, enabling flexible, low‐noise, and highly efficient manipulation of the vector beams.

## 2 Theoretical Framework and Operational Mechanism

### 2.1 Generation of Vector Beams Using Metasurfaces

Vector beams exhibit an inhomogeneous polarization distribution across their cross‐section. Metasurfaces enhance the generation versatility of vector beams by meticulously designing the geometry and arrangement of meta‐atoms. When an input beam with a polarization state |*s*_in_〉 interacts with a meta‐atom, it results in an output beam with a modified polarization state |*s*_out_〉, described by the relation |*s*_in_〉$=J|s_{\mathrm{out}}\rangle$. Here, J represents the Jones matrix associated with the meta‐atom, which characterizes the linear optical response of the meta‐atom to the incident light, as defined by^[^ ^39^ ^]^

(1) $$\begin{array}{ccc} & & J=e^{i\psi_{D}}\\ & & \times \;\left[\begin{array}{cc} \cos\left(\frac{\psi_{B}}{2}\right)+i\sin\left(\frac{\psi_{B}}{2}\right)\cos\left(2\psi_{R}\right) & i\sin\left(\frac{\psi_{B}}{2}\right)\sin\left(2\psi_{R}\right)\\ i\sin\left(\frac{\psi_{B}}{2}\right)\sin\left(2\psi_{R}\right) & \cos\left(\frac{\psi_{B}}{2}\right)-i\sin\left(\frac{\psi_{B}}{2}\right)\cos\left(2\psi_{R}\right) \end{array}\right] \end{array}$$

where ψ_D_ denotes the dynamic phase introduced, ψ_B_ signifies the birefringent phase difference between the two eigen‐polarizations, ψ_R_ represents the orientation angle of the meta‐atom (detailed in Section S1.1, Supporting Information). By precisely adjusting these three parameters of the meta‐atom, one can generate a wide range of polarization states in the output light, regardless of the fixed polarization state of the incident light. Through strategic arrangement of the meta‐atoms to create spatially varying distributions of {ψ_D_, ψ_B_, ψ_R_}, the conversion from scalar beams to vector beams with complex polarization distributions can be effectively achieved. Moreover, by incorporating the geometric phase into the transformation process, it becomes possible to design vector beams that exhibit unique polarization profiles tailored to specific input polarization states. This capability enables the generation of highly customized vector beams suitable for advanced optical applications.

**Figure 1:**
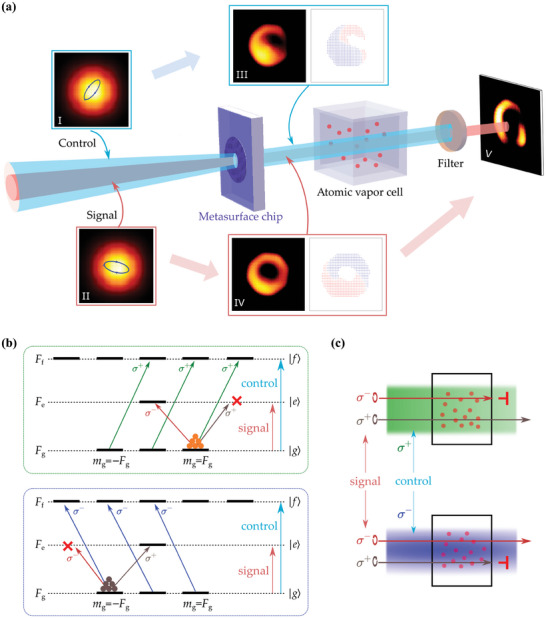
(a) The schematic diagram illustrates intensity modulation in vectorial dual beams produced by combining a single metasurface chip with a thermal atomic vapor cell. Both the input control light (I) and the signal light (II) are Gaussian beams, each displaying unique elliptical polarizations. The corresponding vectorial dual beams (III, IV) generated by the same metasurface exhibit their distinct polarization distributions. The intensity profile of the output signal beam (V) is clipped due to interactions with thermal atoms. (b) The schematic illustrates the atomic energy levels and transitions facilitated by interactions with control and signal lights. In the upper panel, the atomic transition |*g*〉 → |*f*〉 is induced by interaction with σ^+^ polarized control light. This redistributes the atomic population among ground magnetic states, thereby inducing an inaccessible transition |*g*〉 → |*e*〉 for σ^+^ polarized signal light, resulting in circular dichroism within the system. The lower panel presents the opposite scenario, where the system is influenced by the σ^−^ polarized control light. (c) The absorption of the signal beam by atoms is dependent on optical spin when influenced by control light. Specifically, when the control light is σ^+^(σ^−^) polarized, the signal light with σ^−^(σ^+^) polarization is absorbed, whereas the signal light with σ^+^(σ^−^) polarization remains transparent.

As illustrated in **Figure** **1**a, the incident light is an elliptically polarized Gaussian beam that passes through a metasurface chip. Consequently, the polarization profile of the resulting vector beams can simultaneously encompass various elliptically polarized fields. Modifications to the input polarization state lead to corresponding changes in the polarization profiles of the generated beams, as illustrated by the transformations from Figure 1a.I, II to Figure 1a.III, IV. The beam profile and polarization distribution are depicted in Figure 1a.III, IV. By utilizing a metasurface, vector beams can be generated and their properties can be modified by adjusting the polarization state of the input light. However, for a given metasurface structure, the output profile is fixed for each specific polarized input light. In certain practical scenarios, there may be a need to dynamically adjust or modify the profile of the generated beam in response to particular input conditions. To address this necessity, we introduce an atomic ensemble that leverages the mediating role of atoms to shape the signal beam using a control beam.

### 2.2 Atomic Interactions with Dual Light Beams

In Figure 1b, the three‐level hyperfine structure of an atom is depicted, comprising a ground state |*g*〉 and two excited states |*e*〉 and |*f*〉. Each energy level contains multiple degenerate magnetic sublevels, denoted as |*F*_g_, *m*_g_〉, |*F*_e_, *m*_e_〉, and |*F*_f_, *m*_f_〉, respectively. In this notation, {*F*_g_, *F*_e_, *F*_f_} signify magnitudes of the total atomic angular momentum, while {*m*_g_, *m*_e_, *m*_f_} represent the corresponding magnetic quantum numbers. When light interacts with the atom, an electron can absorb a photon and undergo a transition from the ground state |*g*〉 to one of the excited states |*e*〉 or |*f*〉. The selection rules for these transitions are determined by the conservation of angular momentum, which guarantees that only particular combinations of *F* and *m* values are allowed. To be specific, the transition from |*F*_g_, *m*_g_〉 to |*F*_e, f_, *m*_e, f_ = *m*_g_ − 1〉 takes place for left‐circularly polarized light (σ^−^), while the transition from |*F*_g_, *m*_g_〉 to |*F*_e, f_, *m*_e, f_ = *m*_g_ + 1〉 occurs for right‐circularly polarized light (σ^+^). A circularly polarized strong light that is resonant with the transition from |*g*〉 to |*f*〉 acts as the control light and induces a redistribution of the population among the atomic magnetic sublevels within an ensemble. As the system approaches steady state, the majority of atoms occupy the |*F*_g_, *m*_g_ = *F*_g_〉 for σ^+^ polarized light, and |*F*_g_, *m*_g_ = −*F*_g_〉 for σ^−^ polarized light. This polarization‐dependent population redistribution is crucial for controlling the interaction between the signal beam and the atomic ensemble, enabling dynamic adjustments to the vector beam's characteristics.

At this point, a circularly polarized weak light, designated as the signal light and nearly resonant with the |*g*〉→ |*e*〉 transition, is introduced into the system. The polarization state of the control light modulates the population distribution among the ground magnetic sublevels, consequently influencing the absorption of the signal light by the atoms. When the control light is σ^+^ polarized, as shown in the upper panel of Figure 1b, nearly all atoms are found in the |*F*_g_, *m*_g_ = *F*_g_〉 state. Consequently, the signal light with the same σ^+^ polarization remains transparent due to the lack of available transitions for further absorption. In contrast, σ^−^ polarized signal light experiences significant absorption because it can induce transitions from the populated ground state to excited states. Conversely, when the control light is σ^−^ polarized, as illustrated in the lower panel of Figure 1b, the effects on the signal light are reversed. As a result, when the control light interacts with an atomic ensemble, pronounced circular dichroism is exhibited for weak signal light, as shown in Figure 1c. This dichroism stems from the differential absorption of left‐ and right‐circularly polarized signal light, which depends on the polarization of the control light (Figure S8, Supporting Information). For vector beams, this effect results in spatially varying absorption. The atomic vapor interacts distinctly with the σ^−^ and σ^+^ polarized components of the vector beams, leading to beam clipping with spatial modulation of the intensity pattern. The design of metasurface chips is crucial for generating vector beams with precisely customized polarization states. This capability promotes tunable atomic absorption, thereby enhancing the versatility of light field manipulation. In this setup, a vectorial control beam can modulate another vectorial signal beam, allowing for the spatial shaping of the beam profile.

### 2.3 Clipping the Intensity Profile of Signal Light

When vector beams generated by a metasurface chip enter an atomic ensemble, the electric fields of the signal beam **E**_s_ and the control beam **E**_c_ are decomposed based on the atomic circular eigen‐responses in cylindrical coordinates as follows:

(2) $$\begin{array}{cc} E_{s}\left(r,\phi \right) & =A_{s}\left(r,\phi \right)\left[a_{-}\left(r,\phi \right){\hat{e}}_{-}+a_{+}\left(r,\phi \right){\hat{e}}_{+}\right]\\ E_{c}\left(r,\phi \right) & =A_{c}\left(r,\phi \right)\left[b_{-}\left(r,\phi \right){\hat{e}}_{-}+b_{+}\left(r,\phi \right){\hat{e}}_{+}\right] \end{array}$$

where *A*_s_(*r*, ϕ) and *A*_c_(*r*, ϕ) represent the spatial amplitude distributions of the signal and control beams, respectively. The terms *a*_±_(*r*, ϕ) and *b*_±_(*r*, ϕ) denote the normalized components of the respective beams in the right (${\hat{e}}_{+}$) and left (${\hat{e}}_{-}$) circularly polarized bases. In scenarios where both the control and signal lights are vector beams passing through an atomic vapor cell, their interaction shows spatial variability owing to the non‐uniform polarization distributions within the atomic ensemble. In the regions where both the control and signal fields exhibit circular polarization, the transmissivity of the signal light depends on whether their optical spins are antiparallel or parallel. A significant absorption of the signal light occurs when the control and signal fields have opposite circular polarizations. On the contrary, transparency dominates when they have the same circular polarization. Generally, when two beams are either elliptically or linearly polarized, they can be decomposed into σ^−^ and σ^+^ polarized components. This decomposition leads to varying degrees of absorption for the signal light. Consequently, the output intensity of the signal light strongly depends on the spatial polarization distributions of both the signal and control beams. As shown in Figure 1a. V, the output intensity of signal light ($I_{\mathrm{out}}$) can be evaluated by

(3) $$I_{\mathrm{out}}\left(r,\phi \right)\propto A_{s}^{2}\exp\left[-\frac{2\pi \kappa l}{\lambda_{s}}\left(1-S_{s}\cdot S_{c}\right)\right]$$

where λ_s_ is the vacuum wavelength of signal light, κ corresponds to the maximal absorption coefficient for signal and control fields with opposite circular polarizations, *l* is the length of light‐atom interaction, **S**_s_ and **S**_c_ denote the average photon spins of the signal and control lights, respectively, whose magnitudes are given by $S_{s}=a_{+}^{2}-a_{-}^{2}$ and $S_{c}=b_{+}^{2}-b_{-}^{2}$ (detailed in Section S1.2, Supporting Information).

## 3 Design and Experimental Results

### 3.1 Metasurface Design and Fabrication

**Figure 2:**
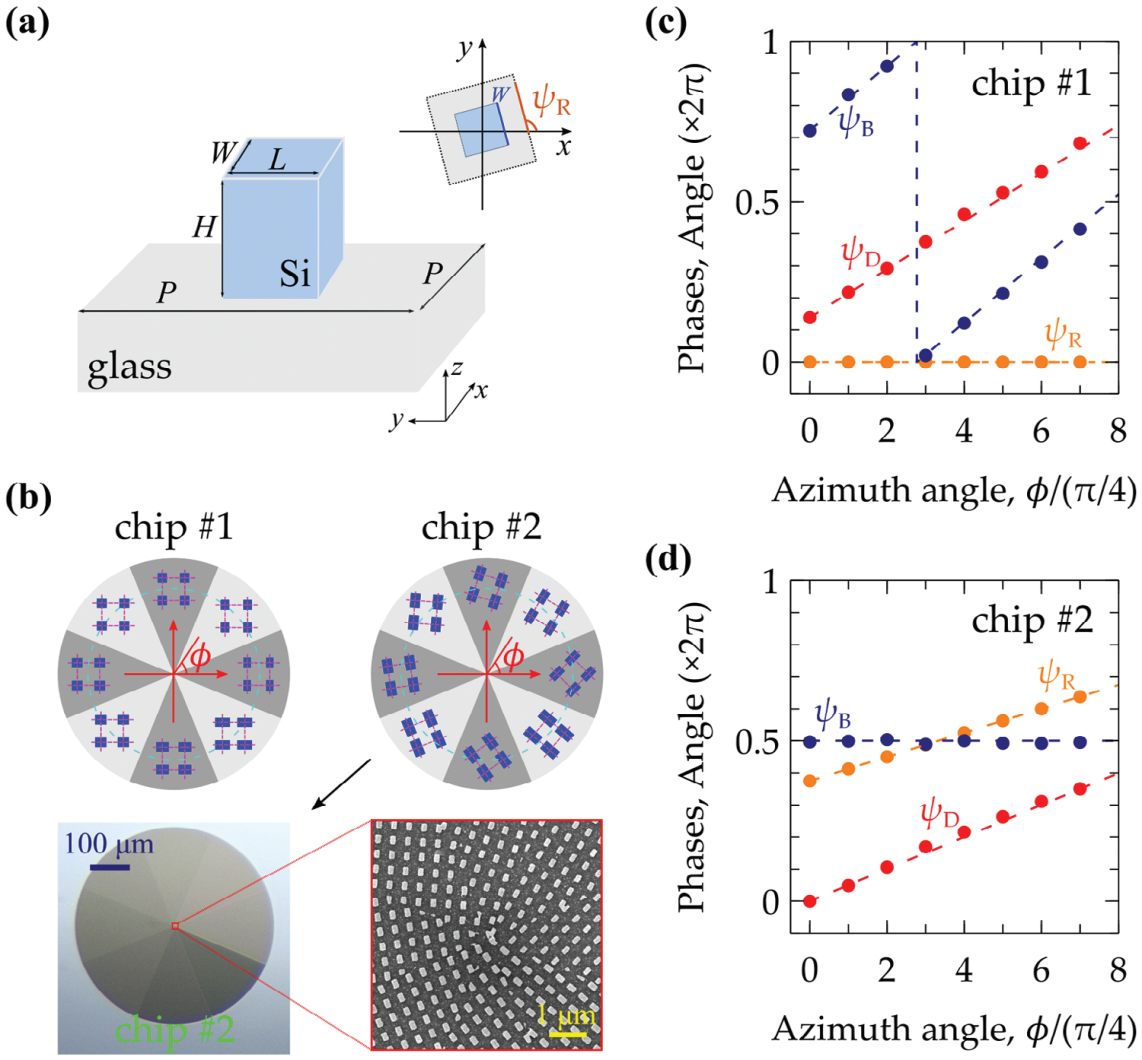
(a) The schemed meta‐atom is composed of a silicon nanofin on a glass substrate. The nanofin has dimensions of length *L*, width *W*, and height *H*. The pitch of the square array is denoted by *P*. The inset shows the rotation of a single meta‐atom at an angle ψ_R_. (b) In the upper panel, the configurations of meta‐atoms in two metasurface chips (#1 and #2) are shown, designed to generate distinct distributions of vector beams. Along the azimuthal axis ϕ, unique set of angular parameters {ψ_D_, ψ_B_, ψ_R_} is utilized in individual sectors for (c) chip #1 and (d) chip #2. In the lower panel of (b), both a photographic image and a scanning electron microscope (SEM) image of chip #2 are displayed.

In this study, we engineered meta‐atoms for a metasurface to modulate the parameters {ψ_D_, ψ_B_, ψ_R_} within the Jones matrix.^[^ ^39^, ^40^ ^]^ As shown in **Figure** **2**a, the metasurface pattern was fabricated on a 1‐mm thick silicon‐on‐glass substrate and arranged in a circular configuration with a diameter of 0.5 mm. To achieve spatially varied polarization profiles for the generated light beams, we segmented the metasurface pattern into eight fan‐shaped sectors, as illustrated in Figure 2b. Each sector contains meta‐atoms of a specific design, all sharing the same dimensions. The meta‐atoms are constructed from silicon nanofins, each with a height (*H*) of 400 nm, varying in width (W) and length (L) from 110 nm to 240 nm. These nanofins are arranged in a square array with a pitch (*P*) of 400 nm, and the tunable {ψ_D_, ψ_B_} are simulated at a wavelength of 780 nm for different nanofin dimensions (Section S1.1, Supporting Information). Furthermore, the meta‐atoms can be oriented at angles ψ_R_ ranging from 0° to 360° according to design requirements.

To manipulate the polarization states with meta‐atoms, structural birefringence must be introduced. This can be achieved by altering ψ_B_ to adjust the birefringence phase difference or tuning ψ_R_ to rotate the eigenresponses, as expressed in Equation (1). In our work, we designed two distinct metasurface chips—denoted as chip #1 and chip #2—each customized to manipulate different parameters along the azimuthal axis ϕ. Specifically, in the design of chip #1, ψ_B_ and ψ_D_ vary with ψ_R_ = 0; in the design of chip #2, ψ_R_ and ψ_D_ vary while keeping ψ_B_ = π$. As shown in Figures 2c and 2d for chip #1 and chip #2, respectively, each symbol at each ϕ represents a unique configuration {ψ_D_, ψ_B_, ψ_R_} employed for an individual sector (Section 1.1, Supporting Information). For these two designs, the eight sectors incorporate diverse silicon nanofin dimensions and arrangements, which are illustrated in the upper panel of Figure 2b. In the lower panel of Figure 2b, both a photographic image and a scanning electron microscope (SEM) image of the fabricated metasurface of chip #2 are presented. By fine‐tuning input light polarization states, the two designs enable the realization of vector beams with doughnut‐shaped and Gaussian‐like intensity patterns under specific input polarization states (Section S2.1, Supporting Information). It is important to note that the performance of the designed metasurface depends on the wavelength. However, due to the small separation between the operation wavelengths of 780 nm and 795 nm, there are no significant differences in the resulting beam patterns or performance (Figure S7, Supporting Information).

### 3.2 Generating Tunable Beam Patterns with Metasurfaces

**Figure 3:**
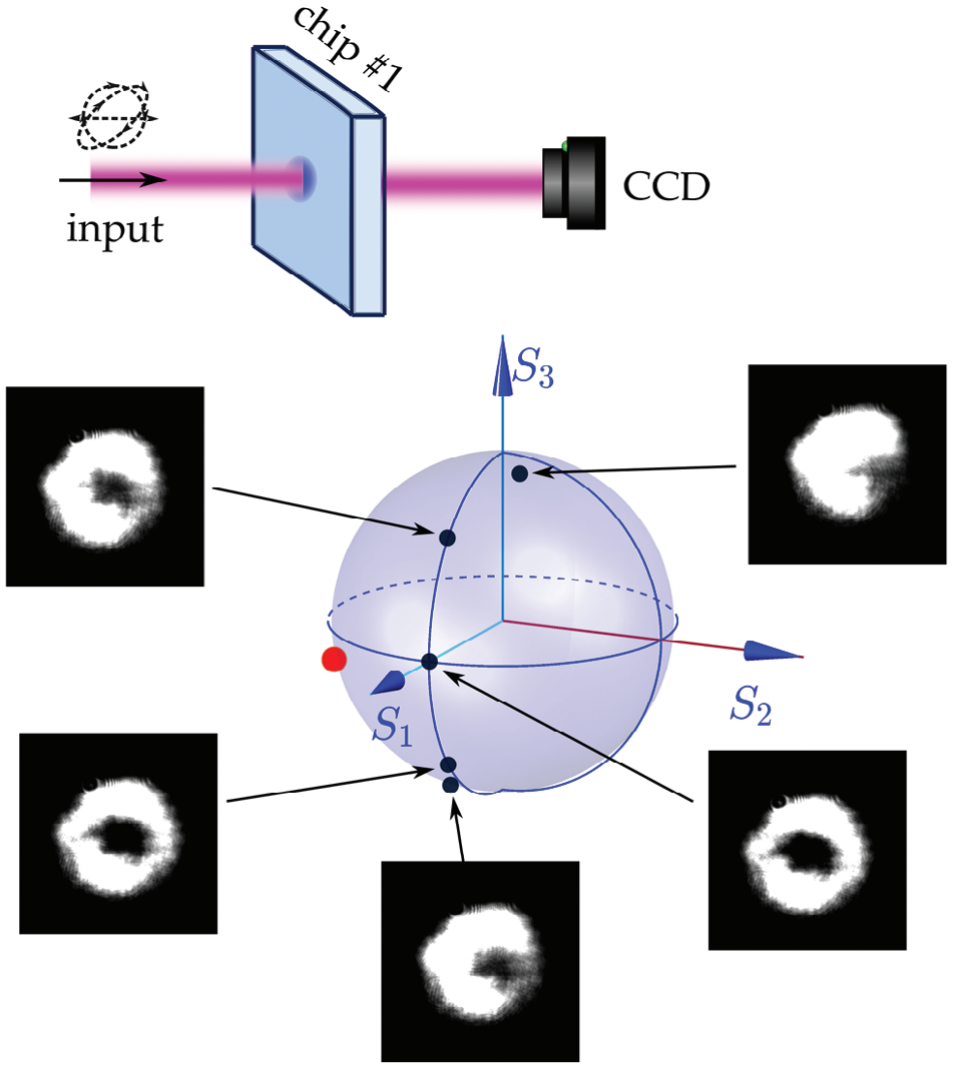
Intensity distribution of the signal vector beam generated by metasurface chip #1 varies with changes in the polarization state of the incident light, as represented on a Poincaré sphere.

By employing a specifically designed metasurface chip, the pattern of the light beam can be adjusted by tuning the polarization of the input light. As depicted in the inset of **Figure** **3**, a Gaussian‐distributed signal beam is directed through chip #1, with its polarization state systematically varied. The resulting output intensity patterns, captured by a CCD camera, are illustrated in Figure 3 for different input polarization states, represented on a Poincaré sphere. The varied patterns effectively highlight the ability to modulate the intensity distribution of the output signal beam by simply adjusting the polarization of the input signal light.

However, it is crucial to note that the output beams often exhibit doughnut or crescent shapes for all other input polarization states, which may be undesirable for certain applications. Moreover, there are scenarios where the polarization states of these signal beams cannot be tuned, necessitating an alternative method for controlling the beam pattern modulation. To overcome this limitation, the study proposes incorporating a control beam to further modulate the signal beams through interactions with thermal atoms. This additional control permits more accurate and versatile modulation of the beam pattern.

### 3.3 Experimental Setup for Beam Shaping Using Atomic Vapor

In the experiment, an atomic ensemble was successfully implemented through the utilization of a vapor cell containing rubidium (Rb) atoms. As is illustrated in the level structure shown in Figure 1b, ^87^Rb atoms were employed. Meanwhile, the *F*_g_ = 2 → *F*_f_ = 3 transition of the D2 line was selected as the |*g*〉 → |*f*〉 transition, and the *F*_g_ = 2 → *F*_e_ = 1 transition of the D1 line was chosen as the |*g*〉 → |*e*〉 transition (detailed in Section S1.2, Supporting Information). In practical scenarios, when atoms transition from excited states back to the ground state, some may de‐excite to the 5^2^S_1/2_(*F* = 1) energy level, thereby exiting the three‐level transition cycles under consideration. To address this issue, we introduced a repump light that resonates with the *F* = 1 → *F*_f_ = 3 transition of the D2 line. This ensures that the atoms are re‐excited and returned to the relevant transition cycles, maintaining their participation in the desired optical processes.

**Figure 4:**
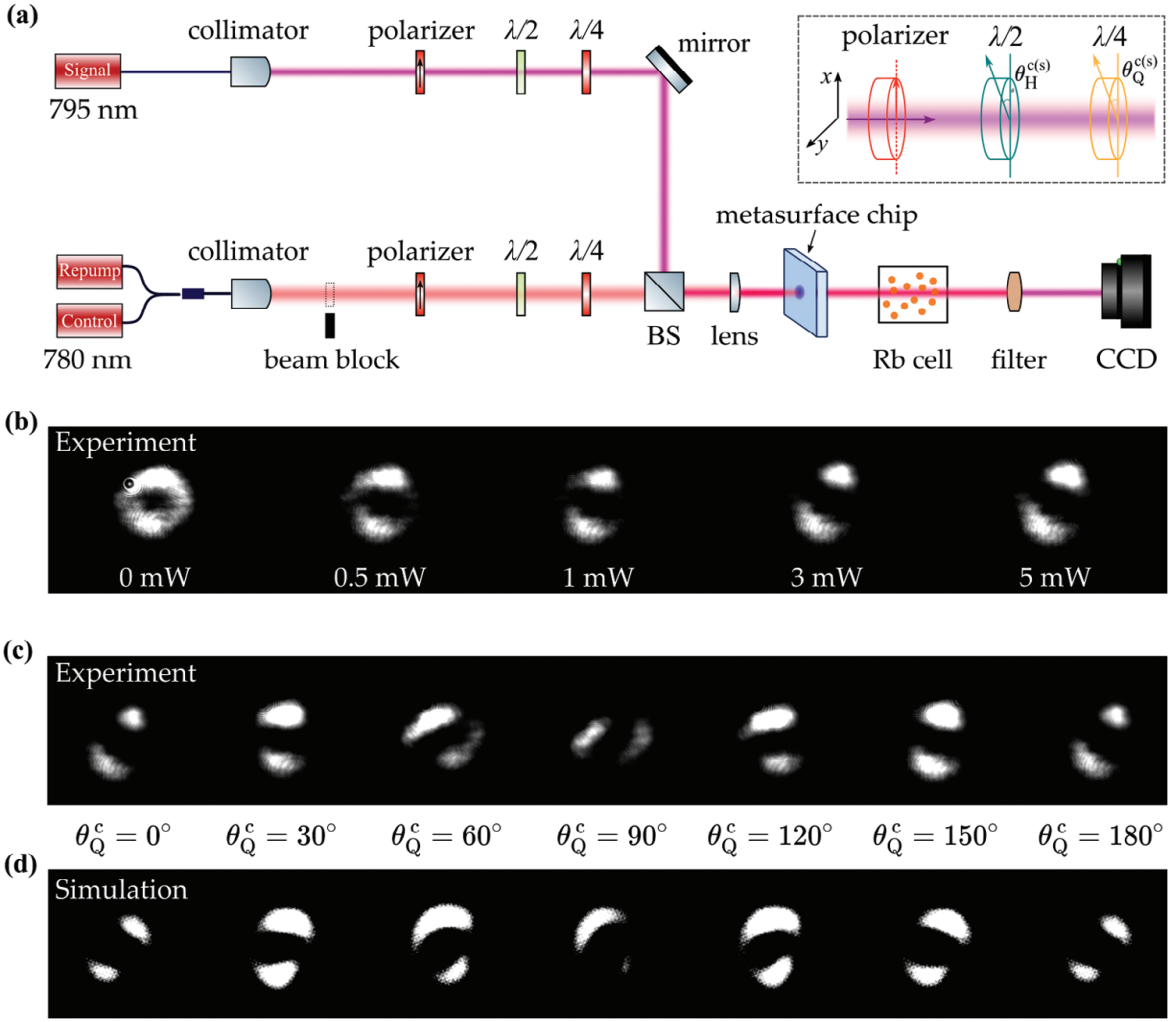
Experimental and simulation results of the intensity distribution for the signal vector beam modulated by the control vector beam, generated using chip #1. (a) Experimental setup with control/repump (red) and signal (pink) light beams. It includes wave plates for regulating polarized light, with an inset diagram showing the rotation angle of each wave plate. A beam splitter (BS) combines the beams, which then pass through a metasurface chip and a Rb vapor cell. A 795 nm narrow bandpass filter is utilized to ensure that solely the signal light is transmitted to a CCD camera. (b) Intensity distribution of the signal vector beam while varying the power of the control incident light from 0 mW to 5 mW. (c) The experimental and (d) simulated results for the intensity distribution of the signal beam with $\theta_{Q}^{c}$ on the control light varying from 0° to 180°, respectively. In the experiment, the powers of signal, control and repump lights are set as 0.04 mW, 5.5 mW and 1 mW, respectively.

The experimental system, as illustrated in **Figure** **4**a, was constructed based on a metasurface chip and an atomic Rb vapor cell to demonstrate the modulation of vector beams. The system includes a 795 nm laser for the signal light and two 780 nm lasers serving as the control and repump lights. The control and repump lights are combined into a single path using a 50:50 fiber coupler and then converted into a free‐space beam via a fiber collimator. The polarization state of the incident light is tuned by a combination of a polarizer, half‐wave (λ/2) plate, and quarter‐wave (λ/4) plate. As illustrated in the inset of Figure 4a, the signal (control) light is initially polarized along the *x*‐axis by the polarizer. To alter the polarization states, the λ/2 plate is aligned at angles $\theta_{H}^{s}$ ($\theta_{H}^{c}$) and the λ/4 plate is positioned at angles $\theta_{Q}^{s}$ ($\theta_{Q}^{c})$ relative to the polarizer. The signal light is combined with the control light via a beam splitter. After optimizing the spot size via a lens, the signal, control, and repump lights are concurrently transmitted across the metasurface chip. By carefully adjusting the incident laser power, we ensure that the control and repump lights populate atoms in their ground state without causing saturation absorption. In this setup, the side length of the cubic vapor cell is 20 mm, and to enhance the atomic density, the temperature of the cell was maintained at 75 °C. At the endpoint of the optical paths, a 795 nm narrow bandpass filter is implemented to block 780 nm lights, thereby precluding interference from control and repump beams on the final output. Consequently, the signal light enters a CCD camera to obtain the intensity pattern of the signal beam.

### 3.4 Results of Beam Shaping Controlled by Light

With this setup, the manipulation of the control light to tailor the profile of the signal beam is demonstrated. To clarify this manipulation, we maintain a constant power of 40 µW and a specific polarization state of the input signal light, denoted by a red circular marker on the Poincaré sphere in Figure 3. Under this configuration, when chip #1 is employed, an initial doughnut‐shaped output intensity distribution is obtained. Figure 4b illustrates the captured intensity pattern of the signal beam as a function of the varying incident power from 0 mW to 5 mW of the control light, with its polarization states set by $\left\{\theta_{H}^{c},\theta_{Q}^{c}\right\}=\left\{20^{\circ },0^{\circ }\right\}$. Upon introducing the control light into the system and gradually increasing its incident power, the initially doughnut‐shaped signal beam bifurcates into two distinct lobes. This transformation is due to inhomogeneous absorption induced by the atomic vapor. The observed inhomogeneity arises from circular dichroism, which results from the interaction of atoms with the distinct vector beams of the signal and control light generated by the metasurface chip.

Further, adjusting the incident polarization of the control beam permits enhanced flexibility in sculpting the intensity profile of the signal beam. In the experiment, the power of the control light was set at 5.5 mW, with its polarization settings configured as $\theta_{H}^{c}=20^{\circ }$ and $\theta_{Q}^{c}$ varied from 0° to 180° (Figure S5, Supporting Information). The intensity distribution of the modulated signal beam is depicted in Figure 4c, where the two distinct lobed shapes rotate due to varying polarizations of the control light. The intensity pattern of the signal beam rotates counterclockwise as $\theta_{Q}^{c}$ varies from 0° to 90° and clockwise as it ranges from 90° to 180°. The observed variation can be directly ascribed to the modulation of atomic absorption by the control vector beam across different incident polarization states (Figure S11, Supporting Information). The simulated results, which are obtained by using Equation (3), are shown in Figure 4d and demonstrate a good match with the experimental results. This consistency not only validates the theoretical model but also highlights the effectiveness of the metasurface chip in realizing controlled and predictable beam manipulation.

**Figure 5:**
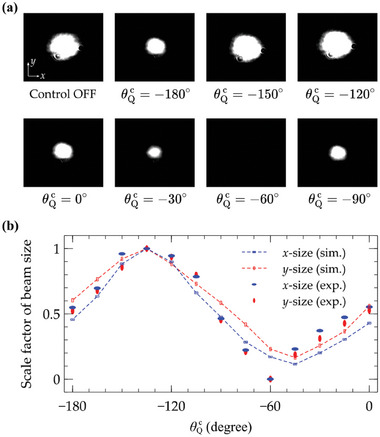
(a) Experimental results of intensity distribution for signal beam modulated by control light, with $\theta_{H}^{c}=0^{\circ }$ and $\theta_{Q}^{c}$ varying from 0° to −180°, under chip #2. (b) Experimental and simulation results of scale factor of beam size versus $\theta_{Q}^{c}$ for the control light. The blue (red) ellipse symbol represents the beam size along the *x*‐axis (*y*‐axis) in the experimental results, while the blue (red) dashed line with a bar signifies the beam size along the *x*‐axis (*y*‐axis) in the simulation results.

Moreover, by utilizing chip #2 and fine‐tuning the polarization state of the incoming signal light, we can produce a beam whose intensity distribution closely approximates a Gaussian profile, as depicted in the first pattern of **Figure** **5**a. Upon activation of the control light, with $\theta_{H}^{c}=0^{\circ }$ and $\theta_{Q}^{c}$ varying from 0° to −180°, the beam size of the Gaussian‐distributed signal beams can be adjusted in response to changes in the control polarization state (Figure S6, Supporting Information). Figure 5b presents the scale factor of the beam size along both the *x*‐ and *y*‐axes, comparing experimental data with calculated results (Section S2.2, Supporting Information). It is clear that as $\theta_{Q}^{c}$ of the control light varies from 0° to −180°, the beam size can be altered by nearly an order of magnitude. This significant modulation suggests that the shape of the signal beam is profoundly influenced and tailored by the control vector beam through the thermal atomic vapor system. The discrepancies observed between the experimental and simulated results of $\theta_{Q}^{c}$ ranging from −60° to 0° are primarily attributed to deviations from the design specifications caused by fabrication imperfections. The absence of a visible spot for $\theta_{Q}^{c}=-60^{\circ }$, as depicted in Figure 5a, is a consequence of the fixed exposure settings of the CCD camera.

## 4 Discussion

Regarding Figures 4c and 4d, there are apparent discrepancies between the simulation and experimental results at certain angles. As explained in Section S1 (Supporting Information), the light field processing workflow has two stages, each with potential inaccuracies. Manufacturing imperfections in Stage I can alter metasurface dimensions and affect meta‐atom parameters, impacting vector beam polarization and causing minor discrepancies between simulations and experimental results, possibly reducing optical pattern precision. In Stage II, we simplified simulations using a phenomenological model [Equation (3)] for clipping results. This model assumes full atomic population in specific magnetic levels under circular polarization, ignoring level populations and coherences. Spatial absorption coefficients may thus be less accurately represented. Current simulations capture main pattern features but may misrepresent finer details. Detailed methodology is in Section S1.2 of the Supporting Information, derived from general atom‐light interactions. However, calculating spatially variant atomic‐induced susceptibility for vector beams remains challenging due to the extensive computations required. Because of these factors, certain discrepancies might emerge between the simulated and the actual performance of the system. In spite of these limitations, the simulations contribute a valuable qualitative comprehension of the overall behavior of the processed light fields.

In previous studies, vector beams interacting with atomic vapors were produced using conventional optical components. This method is intricate and results in a limited variety of polarization distribution patterns, particularly when it comes to generating two vector beams.^[^ ^27^, ^30^, ^31^, ^32^ ^]^ In contrast, metasurfaces offer a versatile and diverse approach to designing generated vector beams.^[^ ^26^, ^34^ ^]^ Additionally, modulating the clipped intensity pattern has typically necessitated the application of a strong magnetic field to adjust atomic energy levels, which is not straightforward. In this work, such modulation is achieved by introducing a control vector beam that alters atomic responses. Importantly, both the control and signal beams can be generated by the same metasurface, enabling functional integration and simplifying the experimental setup.

In this study, we have developed two unique metasurface chip designs to demonstrate the generation and modulation of vector beams. However, the vector beams generated are not confined to these two designs alone. By precisely engineering metasurfaces with specific configurations of meta‐atoms, a diverse distribution of light field outputs can be achieved. This flexibility empowers a multitude of applications through vector beam modulation, such as optical manipulations of micro‐nano particles. Our experimental results reveal that the intensity distribution of the signal beam, when modulated by a control vector beam passing through two distinct metasurface chips, exhibits two characteristic patterns: a dual‐lobed configuration and a Gaussian‐like profile. Modulating the polarization state of the control light allows for the rotation of the dual lobes and dynamic adjustment of the dimensions of the Gaussian‐like profile. This capability enhances the efficiency of trapping and manipulating micro‐nano particles, even at the atomic level. Thus, this dynamic modulation technique possesses considerable potential for applications in atomic trapping, transportation, and associated fields. Furthermore, it also presents promising prospects in the realms of image processing and quantum information.

## 5 Conclusion 

This study introduces a novel hybrid system that combines metasurfaces with thermal atomic vapor to precisely modulate vector beam intensity profiles. A vectorial dual‐beam configuration is introduced, where both the control and signal beams are generated using a single metasurface chip. The inherent flexibility in the design of metasurfaces enables the generation of distinct vector beams through adjustment of their meta‐atom arrangements and geometric configurations. By utilizing the absorption characteristics of atomic vapor, a medium with optical spin‐dependent absorption is constructed, stemming from the control light modifying the atomic population distribution. The circular dichroism feature is utilized to effectively modulate the intensity distribution of the signal beam using the control beam. To demonstrate diverse modulation, we designed and fabricated two metasurface chips capable of generating vector beams with various polarization distributions. By adjusting the power and polarization states of control light, we successfully tailored the intensity profiles of both doughnut‐shaped and Gaussian‐distributed signal beams. The integrated approach for photon‐atom interaction enabled by metasurface technology not only simplifies experimental setups but also paves the way for innovative research and practical applications in advanced optical systems.

## Conflict of Interest

The authors declare no conflict of interest.

## Disclosures

The authors declare no competing financial interests.

## Supporting information

Supporting Information

## Data Availability

The data that support the findings of this study are available from the corresponding author upon reasonable request.
