# Supplementary material for: Thermal Atoms Facilitate Intensity Clipping Between Vectorial Dual‐Beam Generated by a Single Metasurface Chip
Source: Adv Sci (Weinh). 2025 Mar 7;12(17):2416617. doi: 10.1002/advs.202416617 (PMC12061253; doi:10.1002/advs.202416617)
Supplement: Supplementary file 1 — Supporting Information [file ADVS-12-2416617-s001.pdf]

ADVANCED SCIENCE
Open Access

Supporting Information

for *Adv. Sci.*, DOI 10.1002/advs.202416617

Thermal Atoms Facilitate Intensity Clipping Between Vectorial Dual-Beam Generated by a Single Metasurface Chip

*Chen Qing, Jialong Cui, Lishuang Feng and Dengke Zhang**

Supporting Information: Thermal atoms facilitate intensity clipping between vectorial dual-beam generated by a single metasurface chip

*Chen Qing, Jialong Cui, Lishuang Feng, and Dengke Zhang**

School of Instrumentation and Optoelectronic Engineering, Beihang University, Beijing 100191, China

Email: dkzhang@buaa.edu.cn

S1 Modeling and calculations

In this work, we propose a method wherein two vector beams are generated by the same metasurface and subsequently interact with thermal atoms. The entire process can be divided into two stages: in Stage I, the vector beams are produced by the metasurface; in Stage II, these beams interact with the thermal atoms, as illustrated in Fig. S1. The following subsections will detail the working principles of each stage.

S1.1 Stage I: Vector beam generated by metasurfaces

S1.1.1 Jones matrix of meta-atoms

For a lossless-transmission metasurface featuring nanofin arrays in the $x-y$ plane, there exist distinct eigenstates for non-rotational meta-atoms: the x-polarized state $[1,0]^{\mathrm{T}}$ and the y-polarized state $[0,1]^{\mathrm{T}}$. The corresponding eigenvalues are given by $\mu_{1,2} = \exp(\mathrm{i}\varphi_{x,y})$, where $\varphi_{x,y}$ represents the phase shift introduced to $x(y)$-polarized light by the meta-atoms [1, 3]. Consequently, the Jones matrix for this meta-atom, expressed in linear bases, can be written as

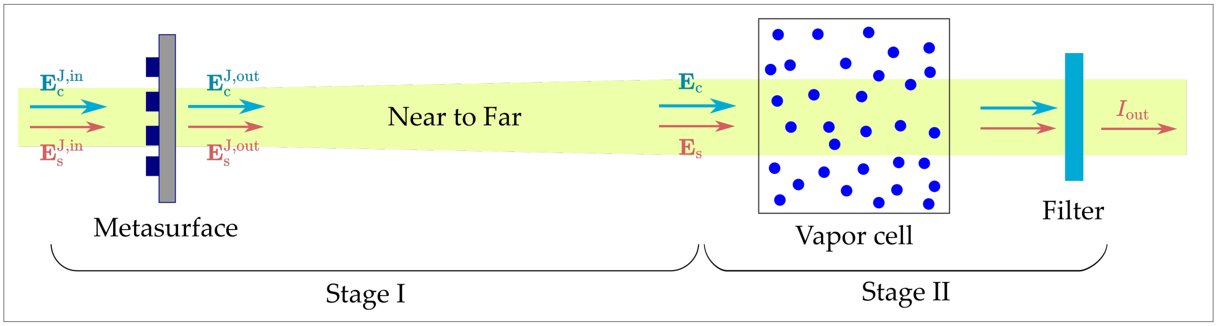

Fig. S1: The entire light flow proceeds through a series of steps: reaching the metasurface, being transformed by it, free propagation, entering the vapor cell and interacting with thermal atoms, exiting the cell, filtering out the control light, and finally reaching the CCD camera. This process can be divided into two main stages: Stage I -generation of vector beams and Stage II -interaction of lights with thermal atoms.

$$\mathbb{J}_0 = \begin{bmatrix} e^{i\varphi_x} & 0 \\ 0 & e^{i\varphi_y} \end{bmatrix}. \tag{S1}$$

When an input beam with polarization state $|s_{\text{in}}\rangle$ passes through a meta-atom, it results in an output beam with polarization state $|s_{\text{out}}\rangle$, which can be described by the relation

$$|s_{\text{out}}\rangle = \mathbb{J}_0 |s_{\text{in}}\rangle. \tag{S2}$$

Furthermore, when a rotation angle ψ_R is introduced to the array, the eigenstates associated with the meta-atoms are transformed as follows: $|q_1\rangle = \mathbb{R}(\psi_R)[1,0]^T$ and $|q_2\rangle = \mathbb{R}(\psi_R)[0,1]^T$, where $\mathbb{R}$ denotes the two-dimensional rotation matrix given by

$$\mathbb{R}(\psi_R) = \begin{bmatrix} \cos(\psi_R) & -\sin(\psi_R) \\ \sin(\psi_R) & \cos(\psi_R) \end{bmatrix}. \tag{S3}$$

Correspondingly, equation (S2) will be revised as follows

$$\mathbb{R}(\psi_R)|s_{\text{out}}\rangle = \mathbb{R}(\psi_R)\mathbb{J}_0\mathbb{R}^{-1}(\psi_R)\mathbb{R}(\psi_R)|s_{\text{in}}\rangle. \tag{S4}$$

Thus for the rotated meta-atom, the Jones matrix $\mathbb{J}$ can be expressed as

$$\mathbb{J} = \mathbb{R}(\psi_R)\mathbb{J}_0\mathbb{R}^{-1}(\psi_R) = \begin{bmatrix} \cos^2(\psi_R)e^{i\varphi_x} + \sin^2(\psi_R)e^{i\varphi_y} & \sin(\psi_R)\cos(\psi_R)\left(e^{i\varphi_x} - e^{i\varphi_y}\right) \\ \sin(\psi_R)\cos(\psi_R)\left(e^{i\varphi_x} - e^{i\varphi_y}\right) & \sin^2(\psi_R)e^{i\varphi_x} + \cos^2(\psi_R)e^{i\varphi_y} \end{bmatrix}. \tag{S5}$$

Further, the Jones matrix $\mathbb{J}$ can be rewritten as

$$\mathbb{J} = e^{i\psi_D} \begin{bmatrix} \cos\left(\frac{\psi_B}{2}\right) + i\sin\left(\frac{\psi_B}{2}\right)\cos(2\psi_R) & i\sin\left(\frac{\psi_B}{2}\right)\sin(2\psi_R) \\ i\sin\left(\frac{\psi_B}{2}\right)\sin(2\psi_R) & \cos\left(\frac{\psi_B}{2}\right) - i\sin\left(\frac{\psi_B}{2}\right)\cos(2\psi_R) \end{bmatrix}, \tag{S6}$$

where $\psi_D \equiv (\varphi_x + \varphi_y)/2$ is the common phase, representing the introduced dynamic phase, and $\psi_B \equiv \varphi_x - \varphi_y$ denotes the birefringent phase difference between transmissions of the two eigenstates. The parameters ψ_D and ψ_B capture the essential phase characteristics:

- ψ_D accounts for the common dynamic phase shift experienced by both eigenstates.
- ψ_B quantifies the birefringent phase difference between the x-polarized and y-polarized light transmissions.

Equation (S6) corresponds to Equation (1) in the main text, describing the Jones matrix for a rotated meta-atom. This formulation provides a comprehensive description of how the polarization states are transformed by a rotated meta-atom, integrating both the intrinsic properties of the meta-atom and the effects of rotation.

S1.1.2 Design configuration of metasurface

For a metasurface constructed from nanofin arrays with varying dimensions, the elements of the Jones matrix are functions of the (x, y) coordinate axes, i.e., $\mathbb{J}$ becomes $\mathbb{J}(x, y)$. For a real input light field with spatial distribution $|s_{\text{in}}(x, y)\rangle$, the output field can be expressed as:

$$|s_{\text{out}}(x, y)\rangle = \mathbb{J}(x, y)|s_{\text{in}}(x, y)\rangle. \tag{S7}$$

Thus, the output field can be locally manipulated point by point through $\mathbb{J}(x, y)$. To generate vector fields, we design the metasurface by spatially varying ψ_B and/or ψ_R while incorporating different ψ_D. In our design, within the polar coordinates (r, ϕ), $\{\psi_D, \psi_B, \psi_R\}$ vary along the ϕ direction but remain constant in the r direction. The transformation described in equation (S7) can be rewritten as

Tab. S1.1: Designed geometric parameters of meta-atoms in chip #1

Meta-atom	Azimuth angle, $\phi/(\pi/4)$							
	0	1	2	3	4	5	6	7
W (nm)	118	140	150	159	169	181	203	240
L (nm)	170	160	158	157	156	154	150	146
ψ_R (deg)	0	0	0	0	0	0	0	0

Tab. S1.2: Designed geometric parameters of meta-atoms in chip #2

Meta-atom	Azimuth angle, $\phi/(\pi/4)$							
	0	1	2	3	4	5	6	7
W (nm)	110	121	129	133	136	137	138	139
L (nm)	196	191	191	198	203	214	226	235
ψ_R (deg)	135	148.5	162	175.5	189	202.5	216	229.5

$$\mathbf{E}^{\mathrm{J,out}}(r,\phi) = \mathbb{J}(\phi)\mathbf{E}^{\mathrm{J,in}}(r,\phi), \tag{S8}$$

where $\mathbf{E}^{\mathrm{J,in}}$ and $\mathbf{E}^{\mathrm{J,out}}$ represent the input and corresponding output fields for the metasurface, constructed from the x- and y-components of the light fields. To simplify the design of $\mathbb{J}(\phi)$, the variations in $\{\psi_D, \psi_B, \psi_R\}$ along ϕ are divided into eight equal fan-shaped sectors, with each parameter remaining constant within a sector. In this work, we designed and fabricated two types of metasurface chips to generate vector beams with distinct polarization distributions:

- **Chip #1**: Varies $\psi_B$and $\psi_D$with $\psi_R = 0$.
- **Chip #2**: Varies $\psi_R$and ψ_D while keeping $\psi_B = \pi$.

By engineering the geometry (length, width, and height of silicon nanofins) and arrangement (divided into eight sectors) of meta-atoms on the glass substrate, we can modulate $\{\psi_D, \psi_B, \psi_R\}$ as described in Equation (1) of the main text, thereby generating various vector beams. In this study, the meta-atoms are constructed from silicon nanofins, each with a height $H = 400$ nm, and dimensions that vary in width (W) and length (L) from 110 nm to 240 nm. These nanofins are arranged in a square array with a pitch $P = 400$ nm and oriented at angles ψ_R ranging from $0°$ to $360°$ according to design requirements. By varying W and L of the meta-atoms, the eigen-responses for x-polarized and y-polarized incident light at a wavelength of 780 nm are simulated and displayed in Fig. S2. Specifically, the phase responses φ_x and φ_y for x-polarized and y-polarized light are shown in Figs. S2a and S2c, respectively. Based on these phase responses, the dynamic phase ψ_D and birefringent phase difference ψ_B of the meta-atoms are calculated and displayed in Figs. S3a and S3b, respectively. The parameters used for chip #1 and chip #2 are marked in these figures. Tables S1.1 and S1.2 summarize the geometric parameters of the meta-atoms for each sector utilized in the two designed metasurface chips. Figures 2(c) and 2(d) in the main text depict the respective values of $\{\psi_D, \psi_B, \psi_R\}$ associated with the two designs. In the experiment, the silicon film growth process and fabrication errors leading to dimensional shifts in the nanofins (typically within 10 nm) can result in deviations from the simulation results. To better match the measurement outcomes, the silicon refractive index and nanofin dimensions can be slightly revised in the simulations.

S1.1.3 Transformation between input and output fields

In the experiment, both the initial input signal and control lights are scalar Gaussian beams that are subsequently transformed into different vector beams by the same metasurface. For each input field, the generated field passing

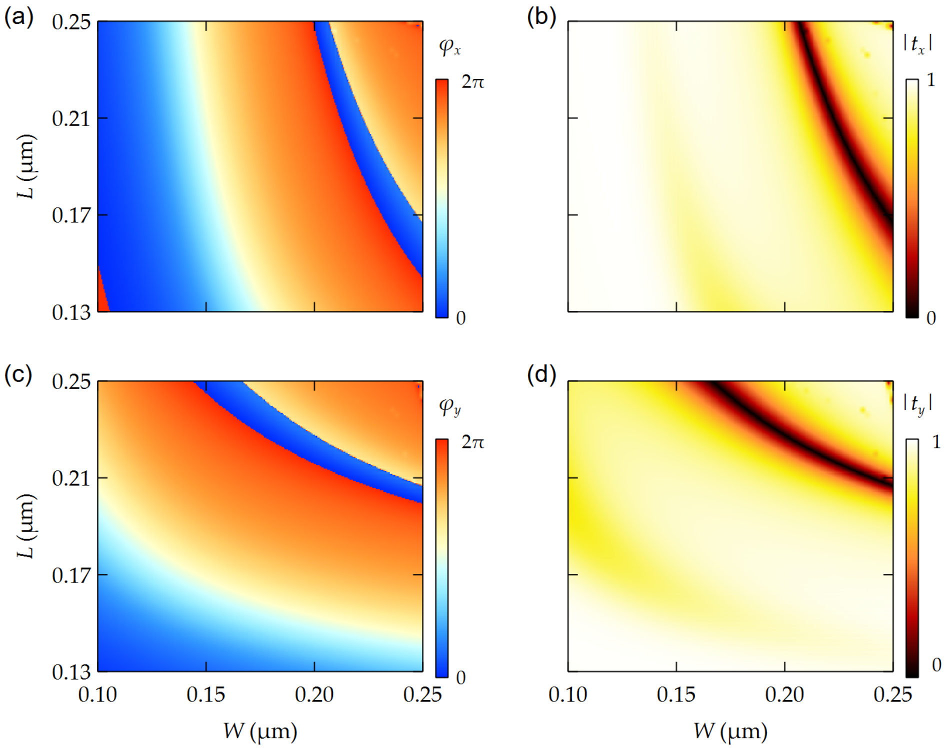

Fig. S2: The eigen-response of meta-atoms to x-polarized incident light at 780 nm, considering variations in width (W) and length (L): (a) φ_x and (b) t_x. Similarly, for y-polarized light, the eigen-response is presented as (c) φ_y and (d) t_y.

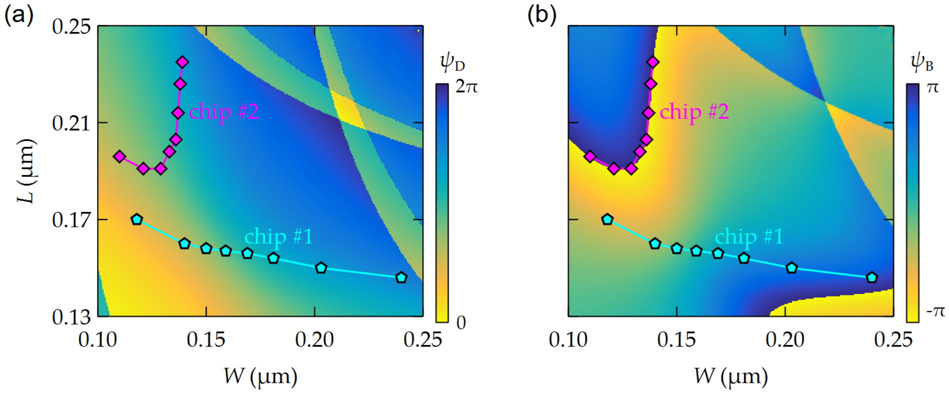

Fig. S3: (a) ψ_D and (b) ψ_B at 780 nm for the meta-atoms with varied width (W) and length (L). The marked points reseprent the parameters used in the design of chip #1 and chip #2.

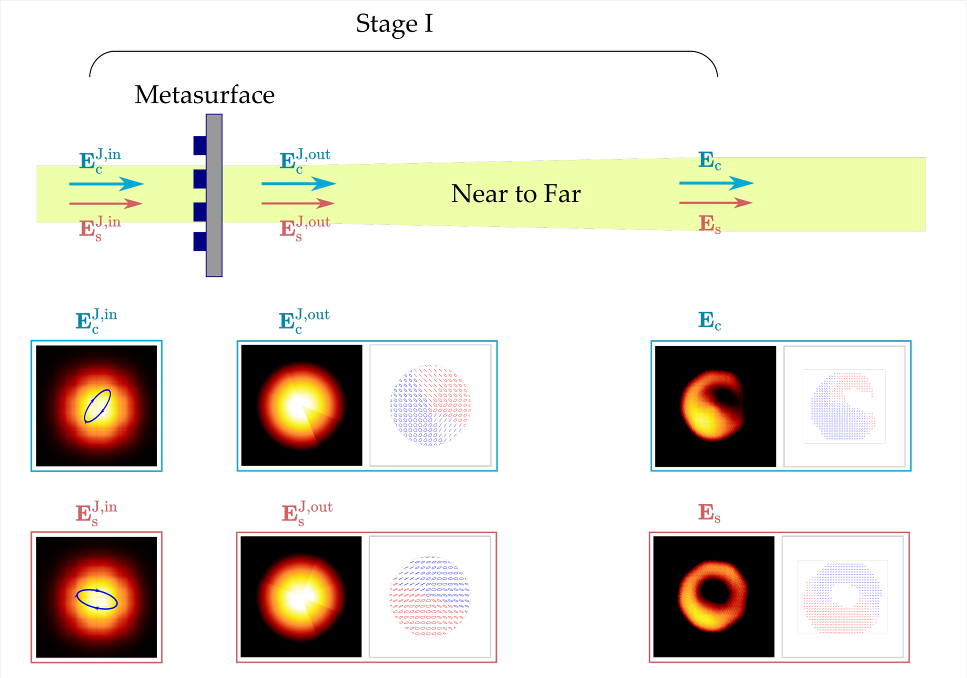

Fig. S4: The vectorial dual-beam is generated by a single metasurface.

through the metasurface is calculated using Equation (S8). The parameters $\{\psi_D, \psi_B, \psi_R\}$ for $\mathbb{J}(\phi)$ can be obtained from the data shown in Figs 2(c) and 2(d) of the main text for chip #1 and chip #2, respectively. Using the generated near-field, the output field is deduced through a typical near-field to far-field transformation. Importantly, the far-field distributions become smooth due to diffraction effects, despite the near-field being comprised of eight discrete and sampled sectorial sources. This transformation redistributes the phase and complex amplitude variations induced by the metasurface, which then interfere and merge as they propagate into the far field. Consequently, well-defined vector beam patterns are achieved. In this work, we require two vector beams to serve as the signal and control lights, respectively. Therefore, we use two input lights, both with Gaussian distributions, denoted as $\mathbf{E}_s^{J,in}$ for the signal light and $\mathbf{E}_c^{J,in}$ for the control light. After passing through the metasurface, these fields are transformed into $\mathbf{E}_s^{J,out}$ and $\mathbf{E}_c^{J,out}$, respectively, as illustrated in Fig. S1. The transformed fields then free-propagate a certain distance and undergo a near-field to far-field transformation. The resulting vector beams are denoted as $\mathbf{E}_s$ and $\mathbf{E}_c$. These beams are subsequently injected into the vapor cell, where they interact with thermal atoms. In Fig. S4, the generation of the vectorial dual-beam using a single metasurface (chip #1) is demonstrated. This figure illustrates how a single metasurface can be utilized to produce two distinct vector beams, each with controlled polarization properties.

S1.1.4 Demonstration of generation of vector beams

The varied design across the eight sectors of metasurface enables to generate the vector beams with varying polarization distributions. By tailoring the generated signal beam to exhibit a doughnut-shaped intensity profile and subsequently modulating the polarization of the input control beam, we effectively achieve modulation of the output signal beam through interaction with thermal atoms (see Figure 4 in the main text). The polarization distributions of the signal and control vector beams along the ϕ axis, as well as their polarization distribution for chip #1, are presented in Fig. S5. The polarization distribution of the output control beam is modified as the incident light's polarization varies at $\theta_H^c = 20^\circ$ and θ_Q^c changes from 0° to 150°.

Figure S6 depicts the polarization distribution of chip #2. The signal vector beam comprises elliptically polarized fields, and its intensity exhibits a Gaussian-like profile. By adjusting the waveplates to set $\theta_H^c = 0^\circ$ and varying θ_Q^c

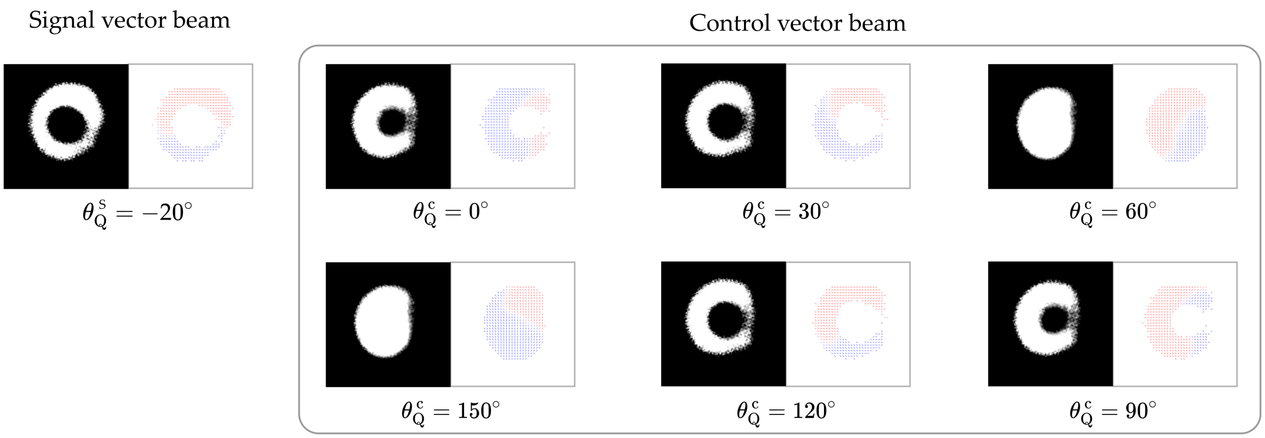

Fig. S5: The intensity patterns and polarization distributions of the signal and control vector beams generated by chip #1 were displayed. The signal beam was set at $\{\theta_H^s, \theta_Q^s\} = \{0°, -20°\}$, while the control beam had a fixed $\theta_H^c = 20°$ with θ_Q^c varying from 0° to 150°.

from $-180°$ to $-30°$, we can manipulate the polarization distribution of the control vector beam. This enables the generation of either linearly or elliptically polarized fields with diverse orientations. For various combinations of control and signal vector beams passing through the thermal atomic vapor, the intensity profile dimension of the output signal beam varies, as illustrated in Figure 5 within the main text.

S1.1.5 Dispersion of the designed meta-atoms

In the experiments, the control and signal lights operate at wavelengths of 780 nm and 795 nm, respectively. Given the dispersion induced by both materials and structure, it is necessary to evaluate the effects of these two wavelengths on the performance of the metasurface. For the small interval between 780 nm and 795 nm, material dispersion can be neglected as it does not cause significant variation in the refractive indices of silicon and glass. Similarly, structural dispersion over such a small wavelength range introduces minimal variances. Figure S7 presents the dynamic phase ψ_D and birefringent phase difference ψ_B at 780 nm and 795 nm for the meta-atoms used in chip #1 and chip #2. According to equation (S6), a constant offset in ψ_D is trivial and does not influence the transformations. Therefore, as illustrated in Figs. S7a,b and S7d,e, the phase differences between 780 nm and 795 nm exhibit little variability. This suggests that the generation of vector beams will not show significant disparities between these two wavelengths, as shown in Figs. S7c and S7f for chip #1 and chip #2, respectively. However, in our simulations for beam clipping, the wavelength-dependent responses of both the control and signal beams are utilized to accurately generate the corresponding vector beams.

S1.2 Stage II: Theoretical analysis of vector beam interaction with thermal atoms

S1.2.1 Modeling the interactions

In our system, the atoms moving with velocity v experience the optical frequencies of the signal and control beams as $\omega_s = \omega_{s0} + k_{s0}v$ and $\omega_c = \omega_{c0} + k_{c0}v$, respectively, where $k_{s0(c0)}$ is the wave number of signal (control) light with the angular frequency of $\omega_{s0(c0)}$. Since the electric field vectors of the signal and control beams are varied in the experiments, and the quantization axis is chosen as the laser beam's propagation direction, the fields of the signal and control beams are given by $\mathbf{E}_s = A_s\,(a_-\hat{\mathbf{e}}_- + a_+\hat{\mathbf{e}}_+)\,\mathrm{e}^{-\mathrm{i}\omega_s t}$ and $\mathbf{E}_c = A_c\,(b_-\hat{\mathbf{e}}_- + b_+\hat{\mathbf{e}}_+)\,\mathrm{e}^{-\mathrm{i}\omega_c t}$, respectively, where $A_{s(c)}$ are the amplitude of signal (control) beams, and $a_\pm(b_\pm)$ are the corresponding normalized components for state of polarization in the spherical bases of $\hat{\mathbf{e}}_{-(+)} = [\hat{\mathbf{e}}_{x(y)} - \mathrm{i}\hat{\mathbf{e}}_{y(x)}]/\sqrt{2}$. Generally speaking, $\hat{\mathbf{e}}_-$ $(\hat{\mathbf{e}}_+)$ corresponds to left-

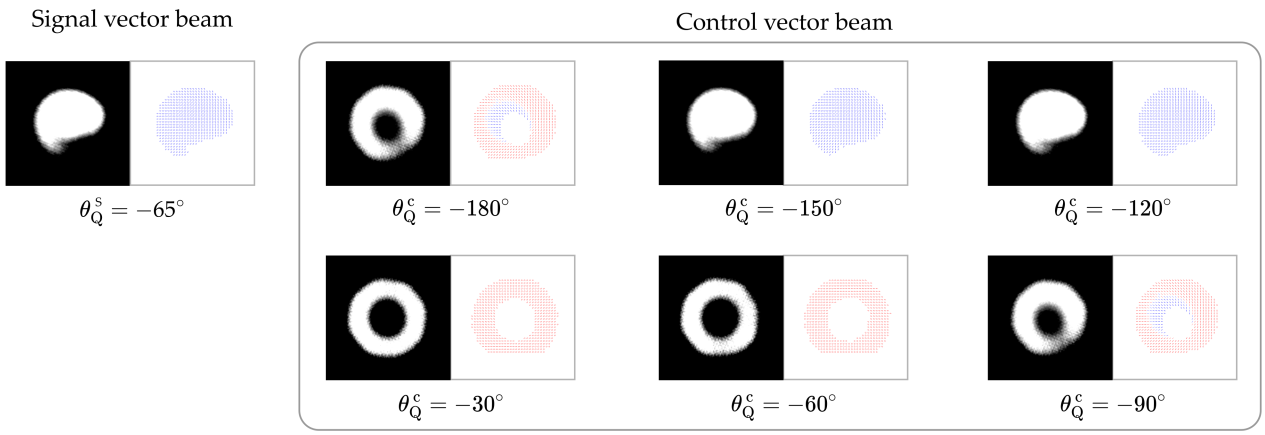

Fig. S6: The intensity patterns and polarization distributions of the signal and control vector beams generated by chip #2 were displayed. The signal beam was set at $\{\theta_H^s, \theta_Q^s\} = \{0°, -65°\}$, while the control beam had a fixed $\theta_H^c = 0°$ with θ_Q^c varying from $-180°$ to $-30°$.

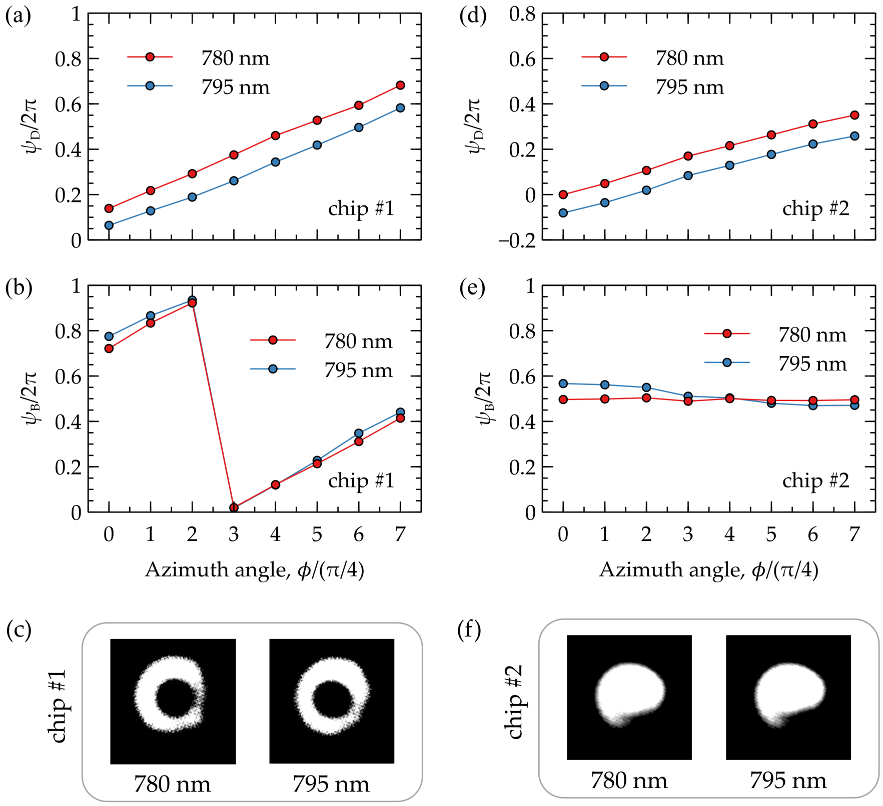

Fig. S7: (a) ψ_D and (b) ψ_B at 780 nm and 795 nm for the meta-atoms utilized in chip #1. (c) An example of beam patterns generated by chip #1 at 780 nm and 795 nm. (d) ψ_D and (e) ψ_B at 780 nm and 795 nm for the meta-atoms utilized in chip #2. (f) An example of beam patterns generated by chip #2 at 780 nm and 795 nm.

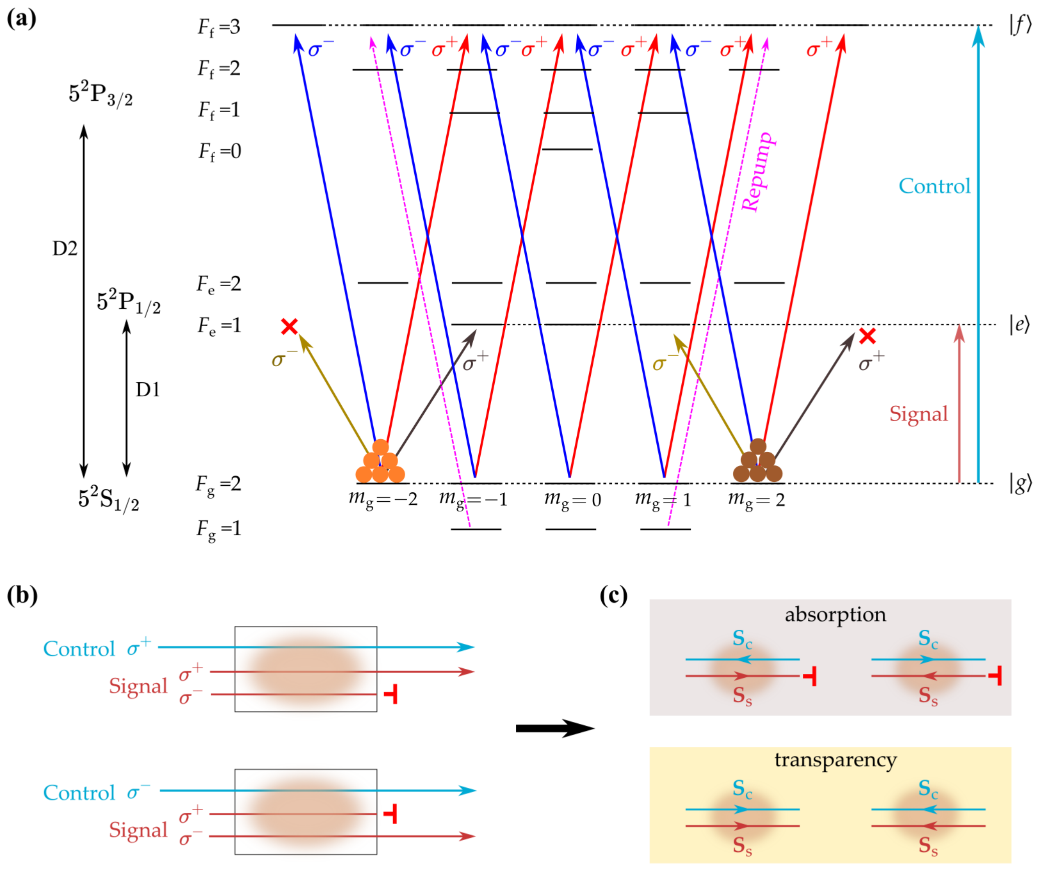

Fig. S8: (a) The atomic energy levels of ^{87}Rb atoms are depicted, where the D2 transition is represented by red (for σ^+ polarization) and blue (for σ^- polarization) solid lines, indicating the control light. Similarly, the D1 transition for signal light is shown in yellow (σ^-) and brown (σ^+) solid lines. The pink dotted lines illustrate the D1 transition associated with the repump light. When the control light is polarized as σ^- (σ^+) and transition $F_g = 2 \rightarrow F_f = 3$, it selectively populates atoms into the magnetic levels $|F_g = 2, m_g = 2\rangle$ $(|F_g = 2, m_g = -2\rangle)$. (b) This non-uniform population prohibits the absorption of a signal light that shares the same polarization , whereas it permits the absorption of orthogonally polarized signal light. (c) The absorption and transparency phenomena correspond to the parallel and anti-parallel alignments of photon spins between the signal and control lights, respectively.

handed σ^- (right-handed σ^+) circularly polarized fields.

The density matrix equation in a rotating frame with frequency ω_c can be written as:

$$\frac{d\rho}{dt} = -\frac{i}{\hbar}[H_0 + V, \rho] + \mathcal{L}, \tag{S9}$$

where ρ is the density operator, H_0 and V are the Hamiltonians of the bare atoms and atom-photon interactions, respectively, and $\mathcal{L}$ denotes the decay process of the system [2, 4]. In this work, the ^{87}Rb atoms are used in the experiment. Figure S1a shows the D1 and D2 transition levels of ^{87}Rb, in which the hyperfine ground state $F_g = 2$ is designated as the ground state $|g\rangle$ and two excited states $F_e = 1$ of D1 and $F_f = 3$ of D2 are donated as $|e\rangle$ and $|f\rangle$, respectively. Considering the corresponding degenerate magnetic states $|F_g, m_g\rangle$, $|F_e, m_e\rangle$, and$|F_f, m_f\rangle$, the atomic Hamiltonian is given by

$$H_0 = -\sum_{m_e} \hbar\left(\delta_c + \Delta_{fe}\right)|F_e, m_e\rangle\langle F_e, m_e| - \sum_{m_f} \hbar\delta_c |F_f, m_f\rangle\langle F_f, m_f|. \tag{S10}$$

In equation (S10), the detuning δ_c is defined as $\delta_c = \omega_c - \omega_{fg}$, where ω_{fg} is the resonance frequency between the states of $|f\rangle$ and $|g\rangle$, and Δ_{fe} is the frequency spacing between the states of $|f\rangle$ and $|e\rangle$.

The atom-photon interaction Hamiltonian is written as

$$\begin{aligned} V = \sum_{m_g} \frac{\hbar}{2} \Big\{ &\Omega_s \left(a_+ e^{i\delta_d t} C^{F_e, m_g+1}_{F_g, m_g} \left|F_e, m_g+1\right\rangle \left\langle F_g, m_g\right| + a_- e^{i\delta_d t} C^{F_e, m_g-1}_{F_g, m_g} \left|F_e, m_g-1\right\rangle \left\langle F_g, m_g\right| + \text{H.C.} \right) \\ &+ \Omega_c \left(b_+ C^{F_f, m_g+1}_{F_g, m_g} \left|F_f, m_g+1\right\rangle \left\langle F_g, m_g\right| + b_- C^{F_f, m_g-1}_{F_g, m_g} \left|F_f, m_g-1\right\rangle \left\langle F_g, m_g\right| + \text{H.C.} \right) \Big\}, \end{aligned} \tag{S11}$$

where $\Omega_{s(c)} = \mu_{eg(fg)} A_{s(c)}/\hbar$ is the Rabi frequency of the signal (control) beam with transition dipole matrix element $\mu_{eg(fg)}$ and $\delta_d \equiv \omega_s - \omega_c$. In equation (S11), $C^{F_{e,f}, m_{e,f}}_{F_g, m_g}$ is the normalized transition strength between the states of $|F_g, m_g\rangle$ and $|F_{e,f}, m_{e,f}\rangle$, and H.C. denotes the Hermitian conjugate. The decay process of the system is given by

$$\mathcal{L} = \sum_{m_g, m_e} \Gamma_{D1} \mathcal{L}_e \left(\left|F_g, m_g\right\rangle \left\langle F_e, m_e\right| \right) + \sum_{m_g, m_f} \Gamma_{D2} \mathcal{L}_f \left(\left|F_g, m_g\right\rangle \left\langle F_f, m_f\right| \right), \tag{S12}$$

where $\Gamma_{D1(D2)}$ is the decay rate of the excited states at D1 (D2) line, and $\mathcal{L}_{f,e}(o) = o\rho o^\dagger - \rho o^\dagger o/2 - o^\dagger o \rho/2$. To obtain the steady-state solutions, the density matrix elements can be expended by

$$\rho = \rho_0 + \rho_1 e^{i\delta_d t}. \tag{S13}$$

In equation (S13), ρ_0 and ρ_1 are responsible for the control and signal responses, respectively. By obtaining the steady-state solutions and combining them with the Maxwell-Boltzmann velocity distribution, we can derive the effective electric susceptibility of the signal light for right/left circular polarized fields ($\sigma^\pm$), which is expressed as

$$\chi_\pm = -N_{Rb} \frac{3\lambda^3}{4\pi^2} \cdot \frac{\Gamma}{\Omega_s} \cdot \frac{1}{\sqrt{\pi} u} \sum_{m=-2}^{2} \frac{C^{F_e, m\pm1}_{F_g, m}}{a_\pm} \int_{-\infty}^{\infty} dv e^{-(v/u)^2} \left\langle F_e, m_g \pm 1 \left| \rho_1 \right| F_g, m_g \right\rangle, \tag{S14}$$

where N_{Rb} is the atomic number density of rubidium in the cell and $u = \sqrt{2k_B T/M}$ is the most probable speed (T: temperature of the cell, M: mass of an atom). Furthermore, the effective complex refractive index of the atomic vapor can be determined as $\tilde{n}_\pm = \sqrt{1 + \chi_\pm}$. The absorption coefficient of the atomic vapor system is given by the imaginary part of the refractive index, denoted as $\kappa_\pm = \text{Im}(\tilde{n}_\pm)$.

S1.2.2 Spatially varied absorption coefficients

Vectorial dual beams display a non-uniform polarization distribution across both the signal and control beams. Consequently, the electric field vectors $\mathbf{E}_s$ and $\mathbf{E}_c$ vary at each point along the beam profile, leading to a spatially varying

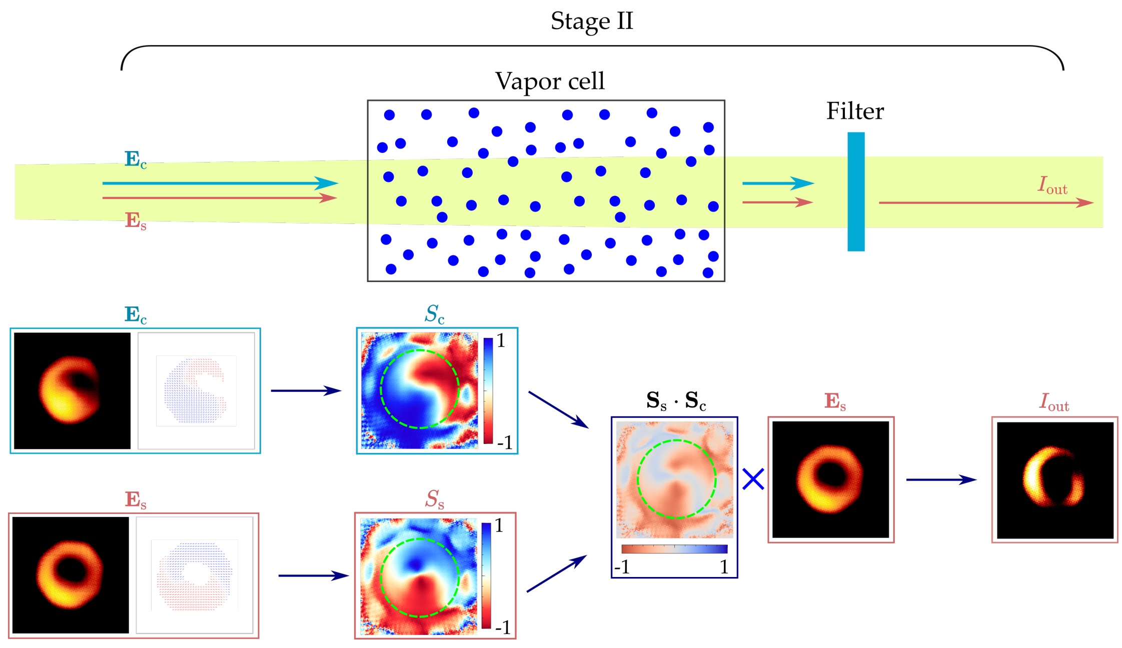

Fig. S9: Intensity clipping between control and signal vector beams is achieved using atomic vapor.

$\chi_\pm$, which in turn gives rise to a spatially variant $\kappa_\pm$. Generally, the intensity profile of a signal beam passing through a vapor cell can be expressed as

$$I_{\text{out}}(r, \phi) \propto \left| A_s \left(a_+ \mathrm{e}^{-\kappa_+ k_{s0} l} + a_- \mathrm{e}^{-\kappa_- k_{s0} l} \right) \right|^2, \tag{S15}$$

where l is the length of light-atom interaction. It should be impressed that A_s, a_+, $\kappa_\pm$ are all functions of r and ϕ. Solving the density matrix equation spatially poses significant computational challenges due to its extensive computational requirements.

As shown in Fig. S8a, the control vector beam modulates the atomic population and induces circular dichroism within the system. Phenomenologically, when the control light's polarization is in either the σ^+ or σ^- circular polarized state, the transition for a signal light with the same circular polarization (σ^+ or σ^-) is prohibited, leading to $\kappa_\pm$ approaching zero. This indicates minimal absorption when the signal and control share the same circular polarization. Conversely, maximal absorption occurs when the signal and control exhibit opposite circular polarizations, as depicted in Fig. S8b. In summary, minimal absorption occurs when the photon spins of the signal and control lights are parallel, whereas maximal absorption is observed when their spins are anti-parallel, as illustrated in Fig. S8c. Taking into account these assumptions and considerations, the output beam intensity can be expressed as

$$I_{\text{out}}(r, \phi) \propto A_s^2 \mathrm{e}^{-\kappa(1-\mathbf{S}_s \cdot \mathbf{S}_c) k_{s0} l}, \tag{S16}$$

where $\mathbf{S}_s$ and $\mathbf{S}_c$ is the average photon spins of signal and control lights, and their magnitudes can be given by $S_s = a_+^2 - a_-^2$ and $S_c = b_+^2 - b_-^2$, respectively. And $\kappa = \max(\kappa_\pm)$ represent the maximal absorption coefficient at the anti-parallel configuration. Utilizing this equation (S16), one can calculate the intensity clipping of a vector beam in a vectorial dual-beam interaction with thermal atomic vapor.

S1.2.3 Demonstration of intensity clipping between control and signal vector beams

In Fig. S9, the process flow of intensity clipping between control and signal vector beams is demonstrated. The two vector beams generated by a metasurface (as shown in Fig. S4) are injected into a vapor cell where they interact

with thermal atoms. According to equation (S16), the average photon spins for the vector beams (S_c and S_s) can be calculated. Specifically, the dot product $\mathbf{S}_s \cdot \mathbf{S}_c$ of the photon spins for the signal and control beams is obtained, as illustrated in Fig. S9. Considering the final output of the signal beams (after passing through a filter), the shaped pattern can be calculated using equation (S16). This allows for control and shaping of the beam's intensity profile based on the interaction with the atomic vapor.

S2 Experimental results

S2.1 Characterize the generated vector beams

To verify the inhomogeneous nature of the vector beam's polarization distribution, the light field is filtered through a linear polarizer that is rotated and then captured by a CCD camera, as illustrated in Fig. S10a. The experimental results for chip #1, shown in Fig. S10b, demonstrate the varying intensity patterns observed at different angles of the polarizer. These patterns indicate the presence of multiple polarization states within the beam. Similarly, for chip #2, the results shown in Fig. S10c also reveal these variations, confirming the complex polarization structure of the generated vector beams. The observed changes in intensity patterns with different polarizer angles confirm that the light field exhibits the characteristics of a vector beam, where the polarization state varies spatially across the beam profile. By comparing these experimental results with simulations, we can validate that the designed metasurface successfully generates the desired vector beams. The close agreement between the experimental observations and theoretical predictions confirms the effectiveness of the metasurface design in producing vector beams with controlled and inhomogeneous polarization distributions.

S2.2 Clipping vector beams via thermal atoms

In our experiment, we established a vector beams modulation system utilizing rubidium atoms (^{87}Rb and ^{85}Rb) enclosed within a $20 \times 20 \times 20\,\mathrm{mm}^3$ glass vapor cell. In order to augment the atomic vapor density, a ceramic heater is employed to elevate the cell's temperature to 75°C. Figure S1 illustrates the D1 and D2 transition lines for ^{87}Rb. The frequencies of the control and repump lights correspond to the $F_g = 2 \to F_f = 3$ and $F_g = 1 \to F_f = 3$ transitions of the D2 line, respectively. Meanwhile, the signal light is associated with the $F_g = 2 \to F_f = 1$ transition of the D1 line. To guarantee a stable and precise laser output, we employ two saturation absorption spectrum stabilization modules and a wavelength meter to lock the control, repump, and signal lights to their respective transitions. The control light with $\sigma^-(\sigma^+)$ circularly polarized field, significantly enhances the atomic population at the magnetic level $|F_g = 2, m_g = 2\rangle$ $(|F_g = 2, m_g = -2\rangle)$. Currently, transition $F_g = 2 \to F_e = 1$ are prohibited for signal lights with the same $\sigma^-(\sigma^+)$ polarization. However, such transitions are permitted when the signal light exhibits the opposite σ^+ (σ^-) polarization. This configuration enables the system to exhibit bidirectional chromatism, wherein the intensity distribution of the output signal light is modulated through adjustments to the control light.

The vector beams, generated by a metasurface chip and characterized by distinct polarization distributions for both control and signal lights, are incident upon the atomic vapor cell. By employing the control vector beam to modulate the signal vector beam, the clipping of the signal vector beam becomes increasingly diverse due to varying polarization distributions. In our experiment, we manipulated the waveplates' orientations to modulate the polarization state of the incoming light, thereby generating a characteristic doughnut-like intensity profile for the signal vector beam using chip #1. Furthermore, we set θ_H^c in the control light path to $\{20°, 40°, 60°\}$ and then varied θ_Q^c from 0° to 180°. As shown in Fig. S11, the intensity distribution of the signal beam indicates that the profile bifurcates into two halves, which rotate in response to changes in the polarization of the control light. Figure 5 in the main text illustrates that the signal beam dimension is modulated when employing chip #2, as a result of spatially dependent atomic absorption influenced by the control vector beam. Figure S12 delineates the experimental and simulated outcomes pertaining to the intensity distribution of the signal beam as the incident polarization state of the control light is varied by θ_Q^c from 0° to −180°.

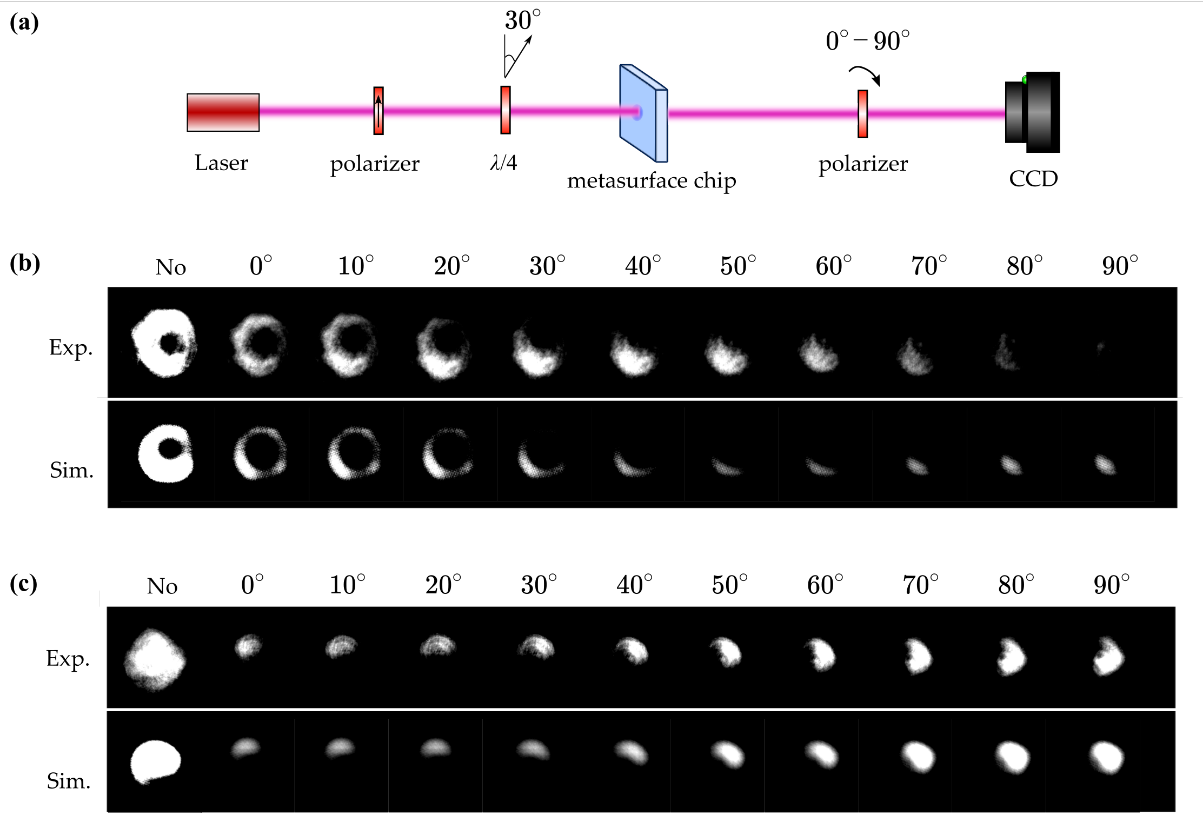

Fig. S10: Characterize the vectorial feature of the generated beams by metasurfaces. (a) Experimental setup for measuring the intensity pattern of vector beams generated by metasurfaces. The input light is Gaussian distributed with elliptical polarization states controlled by a polarizer and a quarter waveplate. The transmitted light from the metasurface passes another polarizer before reaching the CCD camera, and the axis of this polarizer is rotated from 0° to 90°. (b) Using chip #1, the captured intensity patterns without the polarizer and with the polarizer axis from 0° to 90° are shown in the upper panel. The simulated results are presented in the lower panel. (c) Using chip #2, the captured intensity patterns without the polarizer and with the polarizer axis from 0° to 90° are shown in the upper panel. The simulated results are presented in the lower panel.

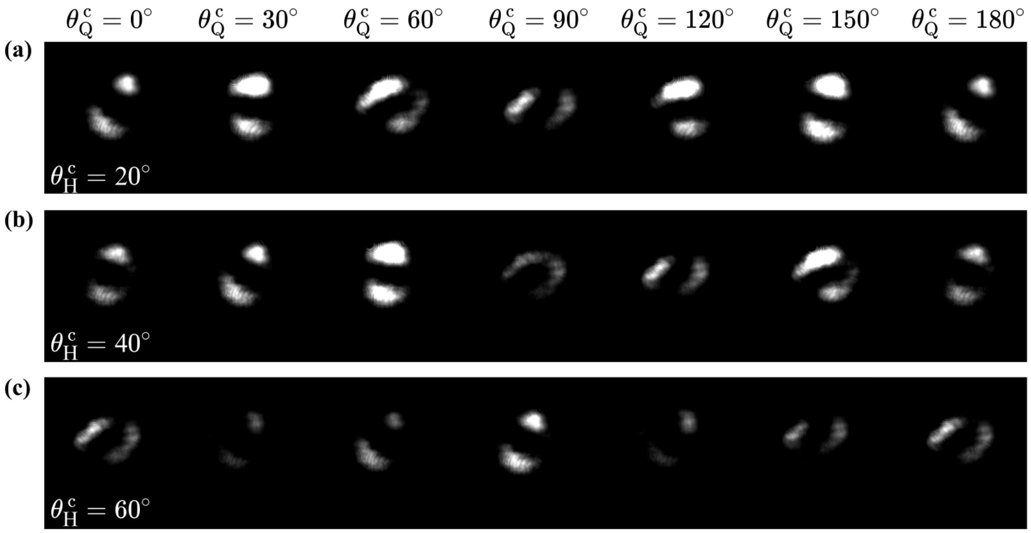

Fig. S11: Using chip #1, experimental results for the intensity distribution of signal beam passing through thermal vapor. The control light was set with θ_H^c equal to (a) 20° (b) 40° (c) 60° while θ_Q^c varied from 0° to 180°.

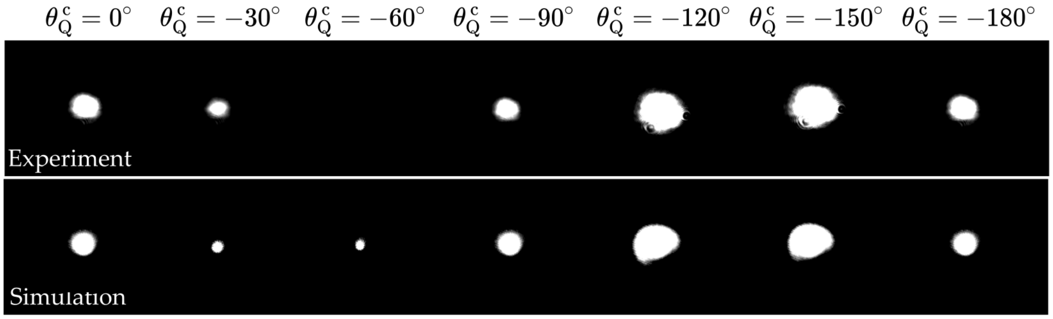

Fig. S12: Using chip #2, experimental and simulation results for the intensity distribution of signal beam passing through thermal vapor. The control light was set with θ_H^c equal to 0° while θ_Q^c varied from 0° to −180°.

References

[1] J. C. Gutiérrez-Vega. Pancharatnam-Berry phase of optical systems. *Opt. Lett.*, 36(7):1143, 2011.

[2] X.-X. Hu, Z.-B. Wang, P. Zhang, G.-J. Chen, Y.-L. Zhang, G. Li, X.-B. Zou, T. Zhang, H. X. Tang, C.-H. Dong, et al. Noiseless photonic non-reciprocity via optically-induced magnetization. *Nature communications*, 12(1):2389, 2021.

[3] D. Zhang, X. Feng, and Y. Huang. Orbital angular momentum induced by nonabsorbing optical elements through space-variant polarization-state manipulations. *Phys. Rev. A*, 98(4):043845, 2018.

[4] D. Zhang and C. Qing. Tailoring sub-doppler spectra of thermal atoms with a dielectric optical metasurface chip. *Laser Photonics Rev.*, 18(7):2301276, 2024.
